# Ginsenoside Rg3 promotes regression from hepatic fibrosis through reducing inflammation-mediated autophagy signaling pathway

**DOI:** 10.1038/s41419-020-2597-7

**Published:** 2020-06-12

**Authors:** Xiangxiang Liu, Xiaojie Mi, Zi Wang, Ming Zhang, Jingang Hou, Shuang Jiang, Yingping Wang, Chen Chen, Wei Li

**Affiliations:** 1 College of Chinese Medicinal Materials, Jilin Agricultural University, 130118 Changchun, China; 2 National & Local Joint Engineering Research Centre for Ginseng Breeding and Development, 130118 Changchun, China; 3 College of Medicine, Jilin University, 130021 Changchun, China; 4 School of Biomedical Sciences, The Universityof Queensland, Brisbane, QLD 4072 Australia

**Keywords:** Pharmacodynamics, Liver fibrosis

## Abstract

Inflammation and autophagy occur during hepatic fibrosis development caused by various pathogens, and effectively curbing of autophage may delay the occurrence of hepatic fibrosis. The current study aimed to unravel the inhibitory effects of Ginsenoside Rg3 (G-Rg3) on inflammation-mediated hepatic autophagy to curb hepatic fibrosis caused by thioacetamide (TAA)-induced subacute and chronic hepatic injury. TAA is mainly metabolized in the liver to cause liver dysfunction. After intraperitoneal injection of TAA for 4 or 10 weeks (TAA-chronic mouse models), severe inflammatory infiltration and fibrosis occurred in the liver. Treatment with G-Rg3 alleviated hepatic pathological changes and reversed hepatic fibrosis in the TAA-chronic models with decreased deposition of collagen fibers, reduced expression of HSCs activation marker (α-SMA), and reduced secretion of profibrogenic factors (TGF-β1). G-Rg3 decreased expressions of autophagy-related proteins in mice of TAA-chronic models. Notably, G-Rg3 inhibited the survival of activated rat hepatic stellate cells (HSC-T6), but had no cytotoxicity on human hepatocytes (L02 cell lines). G-Rg3 dose-dependently inhibited autophagy in vitro with less expression of p62 and fewer LC3a transformation into LC3b in inflammatory inducer lipopolysaccharide (LPS)-induced rat HSC-T6 cells. Furthermore, G-Rg3 enhanced the phosphorylation of phosphatidylinositol 3-kinase (PI3K) and protein kinase B (Akt) in vivo and in vitro. Besides, mTOR inhibitor Rapamycin and PI3K inhibitors LY294002 were employed in LPS-treated HSC-T6 cell cultures to verify that Rg3 partially reversed the increase in autophagy in hepatic fibrosis in vitro. Taken together, G-Rg3 exerted anti-fibrosis effect through the inhibition of autophagy in TAA-treated mice and LPS-stimulated HSC-T6 cells. These data collectively unravel that G-Rg3 may serve a promising anti-hepatic fibrosis drug.

## Introduction

Chronic inflammation in liver is an immune response that persists for months. Inflammation, tissue remodeling, and tissue repairing processes coexist during the chronic inflammation. This inflammation may be induced by several insults, including hepatitis virus infection, excessive alcohol intake, hepatic toxins, autoimmune reactions, and metabolic disorders^1,2^. Regardless of etiology, chronic liver inflammation induces hepatic fibrosis that eventually leads to cirrhosis and hepatocellular carcinoma as the 12th leading cause of death in the US^3^, accounting for 32,000 deaths in the US and more than 1 million deaths each year worldwide^4^. Thioacetamide (TAA), as a potent hepatotoxin, was used to produce the experimental model of hepatic fibrosis and cirrhosis in rodents^5,6^. Compared to classical carbon tetrachloride (CCl4), bile duct ligation (BDL), and nonalcoholic fatty liver disease (NAFLD) models, TAA represented more similarity to cirrhosis caused by human viral hepatitis in hepatic fibrous morphology, hemodynamics, and biochemistry^6,7^. Prolonged intraperitoneal TAA injections result in its high mortality limiting its widespread usage^6,8^. In this research, we modified method to produce chronic inflammation-related hepatic fibrosis mouse model.

Hepatic fibrosis, as a benign stage of cirrhosis, may be reversible to normal when those underlying etiological agents are withdrawal^9–11^. The majority of hepatocellular carcinoma (HCCs) occur following severe fibrosis or cirrhosis^12^. The pivotal event in preventing the progression of hepatic fibrosis is to reduce the activation and transition of hepatic stellate cells (HSCs) into myofibroblasts (MFs) or to promote HSCs’ senescence and apoptosis^13^. Quiescent HSCs exist in the space of Disse (sinusoidal endothelial cell layer), accounting for 5–8% of total cells in the liver, and may possess regulation-like effects on hepatic vasculature system^14^. Activated HSCs cause strong cell proliferation and dramatic changes in their stellate morphology, and meanwhile, are accompanied by lipid deposition, degradation imbalance of extracellular matrix (ECM), and over-secretion of HSC-specific marker protein alpha-smooth muscle actin (α-SMA)^15^. A study has shown that activated HSCs migrated with the infiltration of leukocytes into the damaged area in the liver^16^, whereas inflammatory cells evoked hepatic inflammation^17^ and secreted proinflammatory and profibrotic factors, including transforming growth factor β1 (TGF-β1) and platelet derived growth factor (PDGF) to induce activation of myofibroblasts^18^. It was reported that TGF-β1 activated HSCs, which formed an autocrine-positive feedback loop aggravating hepatic fibro-genesis^19,20^. Autophagy is recognized as cell self-digestion behavior. When autophagy occurs, autophagic lysosomal flux increases to promote dysfunctional organelle, excessive catabolism, misrecognition, or misfolded proteins to metabolize quickly, which then further promotes HSCs activation^21,22^. Recent studies demonstrated that exogenous administration of inflammation inducers lipopolysaccharide (LPS) activated TGF-β1 signaling through promoting autophagy, which aggravated eventually the activation of HSCs^21^. Herein, we aim to search for an appropriate therapy strategy to attenuate hepatic fibrosis through regulating autophagy process and ameliorating chronic inflammation in the liver.

So far, there is no specific anti-fibrosis medicine. Silybin was used but caused obvious adverse events during prolonged and high dosage uses^23,24^. Ginsenoside Rg3 (G-Rg3), as one of protopanaxadiol saponin (PPD) components, was relatively rich in red ginseng (*Panax ginseng* C.A Meyer) and gained excellent reputation for its medicinal properties in immunomodulation, anti-fatigue, myocardial protection, antidiabetic, and anticancer^25^. Our previous work revealed that G-Rg3 exerted an anti-apoptosis effect on hepatocytes in drug-induced acute hepatic injury as a potential hepatoprotective agent^26^. However, whether G-Rg3 exerts unique regulatoin on activated HSCs remains unknown. Here in the present study, we explored the effect of G-Rg3 on hepatic fibrosis caused by chronic inflammation in TAA-treated mice or LPS-stimulated HSC-T6 cells. Further, we studied the role of autophagy in the hepatic fibrosis process and aimed to unravel the molecular mechanism of G-Rg3 on hepatic fibrosis.

## Materials and methods

### Regents and chemicals

TAA (purity ≥ 99.0%) was purchased from Sigma-Aldrich (St. Louis, MO, USA). 20 (R)-G-Rg3 (purity ≥ 98.0%, HPLC) was obtained and qualified as described by our previous study^26^. mTOR inhibitor Rapamycin (Ra) and PI3K inhibitor LY294002 were obtained from Med Chem Express Biotech Co. Ltd. (New Jersey, USA). Catalase (CAT), superoxide dismutase (SOD), glutathione (GSH), malondialdehyde (MDA), H&E staining kit, and Masson staining kit were obtained from Nanjing Jiancheng Bioengineering Research Institute (Nanjing, China). Enzyme-linked immunosorbent assay (ELISA) kit for TGF-β1 was purchased from R&D systems (Minneapolis, MN, USA). Autophagy-related antibodies of LC3 a/b (12741 S), ATG3 (3415 S), ATG5 (12994 S), ATG7 (8558 S), ATG12 (4180 S), ATG16L (8089 S), Beclin-1 (3495), mTOR (2972 S), p-mTOR (2971 S), p-ULK1 (14202), Akt (9272 S), p-Akt (13038), PI3K (4292 S), p-PI3K (422S8), and anti-HRP were from Cell Signaling Technology (Massachusetts, USA). p62 (18420-1-AP), α-SMA (23660-1-AP), TGF-β1 (21898-1-AP), β-actin (60008-1-Ig), and GAPDH (60004-1-Ig) were from Proteintech (Chicago, USA). All other reagents and chemicals, unless indicated, were obtained from Beijing Chemical Factory (Beijing, China).

### Animals

Male-specific pathogen-free (SPF) ICR mice (6–8 weeks old) were bought from the Chang YISI Experimental Animal Co. Ltd. (Changchun, China), and housed under temperature 23 ± 2 °C and 12 h light/dark cycle with ad libitum access to diet, and acclimatized for 1 week prior to the study. All experiment protocol in this study was strictly conducted according to the Guide for Laboratory Animal care and use Committee of Jilin Agricultural University.

#### Experimental design

(I) For induction of subacute hepatic injury, mice were randomly assigned into four groups (*n* = 10, per group): Normal, TAA group, and two G-Rg3 treated groups (5 and 10 mg/kg). The subacute hepatic injury model was established in a dose of 150 mg/kg for TAA according to our preliminary experiments^27^. Intraperitoneal injection with TAA was for 2 weeks before administration orally G-Rg3 at doses of 5 or 10 mg/kg and totally for 4 weeks. The doses of G-Rg3 were appropriately adjusted according to clinical equivalent dose and our preliminary experiments^26,28^.
(II) For induction of chronic hepatic fibrosis, mice were randomly assigned into four groups (*n* = 10, per group): Normal, TAA group, and two G-Rg3 treated groups (5 and 10 mg/kg). In reference to the subacute hepatic injury model, 100 mg/kg was used for the first time, and 50 mg/kg was used in later injections, twice a week, for 10 weeks. G-Rg3 was orally administered with doses of 5 or 10 mg/kg body weight in chronic hepatic fibrosis model for 4 weeks. After 12 h fasting, the mice were subjected to euthanasia. Blood sample was placed at 4 °C for 40 min till next procedures and liver tissues and other organs washed with cooling saline were partially collected in 4% formalin, and others kept at 80 °C for further biochemical analysis.

### Liver function and oxidative stress indicators tests

The AST and ALT levels in serum were measured by commercial kits. Meanwhile, the enzyme activities of SOD and CAT, and content of GSH and MDA were tested to examine changes in oxidative stress levels in TAA-induced liver fibrosis model. Briefly, liver tissue was physically dissociated in 9 volumes of ice-cold 0.9% NaCl and centrifuged twice at 3000 rpm for 10 min at 4 °C, then collected supernatant of liver homogenate. Serum samples were obtained from centrifugation at 1000 × *g* for 10 min at 4 °C. Then, serum samples with substrates or buffer solution were incubated together for 50 min at 37 °C, followed with a color developing agent and measured at a wavelength of 510 nm. BCA kit was used in all involved protein quantification experiments (Beyotime Biotechnology, China). Any abnormal data of the sample (very few maximum and minimum) would be excluded from the group.

### Liver histology examination

Briefly, the liver tissues were immersed in 10% buffered formalin over 24 h embedded in paraffin and cut into a 5-μm-thickness slice. Pathological sections were examined to assess the extent of fibrosis with H&E and Masson’s staining kits. Liver sections were observed with a light microscope (Leica, DM2500, Germany). Representative views of liver sections are performed. In subacute model: the survival mice were re-numbered sequentially, the number of 2nd, 4th, and 6th were chosen for H&E staining. In chronic model: mice were randomly divided and numbered sequentially, the number of 3rd, 6th, and 9th were chosen for pathological observation (including H&E staining, Massons’ staining and Immunofluorescence staining). Masson-stained images were randomly captured from 10 fields (magnification ×100) in three mice/group.

### Cell-culture conditions and drug treatment

Activated phenotype HSC-T6 cells and L02 cells were purchased from ATCC with STR authentication. The rat HSC-T6 cell lines are used for potential therapeutic intervention of activated HSCs owing to it exhibits an activated phenotype and a fibroblast-like morphology after transfection with SV40 sequences^29^. HSC-T6 cells were cultured in Dulbecco’s modified Eagle’s medium (DMEM) with 10% fetal bovine serum (FBS) of defined class and grown in 5% CO_2_ humidified atmosphere at 37 °C. Furthermore, HSC-T6 and L02 cells were seeded in 96-plates and 6-plates at a density of 3 × 10^5^ cells/well and 5 × 10^4^ cells/well, respectively. Before G-Rg3 treatment, cells were grown to approximately 50% confluence, then exposed to G-Rg3 at different concentrations of 0, 2, 4, 8, 16, and 20 µM for different periods of 0, 4, 8, 12, and 24 h. Then the corresponding experiments were carried out. The mTOR inhibitor Rapamycin (Ra) and PI3K inhibitor LY294002 were added 1 h in advance and incubated for 24 h, respectively. For the autophagy induction experiment, the groups are Normal, G-Rg3, Ra (100 nM), G-Rg3+ Ra (100 nM), Ra (200 nM), G-Rg3 + Ra (200 nM), respectively. For the PI3K signaling research experiment, the groups are as follows: Normal, G-Rg3, LPS, LY294002 (20 μM), LPS + G-Rg3, LY294002 + G-Rg3, LPS + LY294002 + G-Rg3. In our current research, all the results carried out in cells from triplicate experiments.

### ELISA

To investigate the fibrogenic situation in TAA-induced liver fibrosis, the concentrations of TGF-β1 both in serum and supernatant of liver homogenate were detected by the R&D system ELISA kit in strict accordance with the manufacturer’s protocols.

### Western blot analysis

Equal amounts of protein obtained from cells or liver tissue samples were run in vertical electrophoresis unit, then transferred to the PVDF membrane (Millipore, USA), and blocked with 5% nonfat milk in TBS-T (TBS + 0.4% Tween-20) for 2 h. Subsequently, the PVDF membrane was incubated with corresponding primary antibodies at a dilution of 1:1000 except GAPDH and β-actin at 4 °C overnight, followed by three washes with TBS-T. HRP-conjugated secondary antibodies were, respectively, used to combine primary antibodies for 2 h, followed by three washes in TBS-T for 8 min each. Finally, blots were incubated with ECL (Proteintech, USA). The intensity of bands was quantified using software Image J.

### Immunofluorescence staining

Briefly, for liver tissues, 5-μm-thickness sections were reprocessed with xylene followed by gradient ethanol. Then, sections were incubated with primary antibody involving rabbit anti-mouse TGF-β1 (1:500) and α-SMA (1:500) in a humidified dark box at 4 °C overnight; they were followed by SABC-dylight448-labeled secondary antibody (Boster, China), incubated for 37 °C, 30 mins. The Nucleus in liver tissues was stained by 4, 6 diamidino-2-phenylindole (DAPI) staining for 5 min, and then examined by a light microscope (Leica, DM2500, Germany).

For cells, HSC-T6 cells were seeded in 6-plates at a density of 5 × 10^4^ cells/well. Prior to G-Rg3 treatment, cells were grown to approximately 50% confluence, then exposed to G-Rg3 for indicated concentration and time, followed by permeabilization of 4% paraformaldehyde with 1% Triton-X100, then incubating with p62 and α-SMA. Other processing steps were similar to the above. Finally, fluorescence was captured by a fluorescence microscope (Leica, DMIL LED, Germany). Related cell trials including western blot and immunofluorescence staining were duplicated three times, respectively.

### Statistical analysis

All data were presented with mean ± standard deviation (mean ± S.D). The difference between groups was analyzed by two-tailed Student’s *t*-test or one-way analysis of variance (ANOVA). For statistical tests, *p < 0.05, *p < 0.01, *p < 0.001 was set to measure the level of significance. GraphPad Prism software package 8.2 was used for figures.

## Results

### G-Rg3 exerted protection against TAA-induced subacute and chronic hepatic injury

**Fig. 1:**
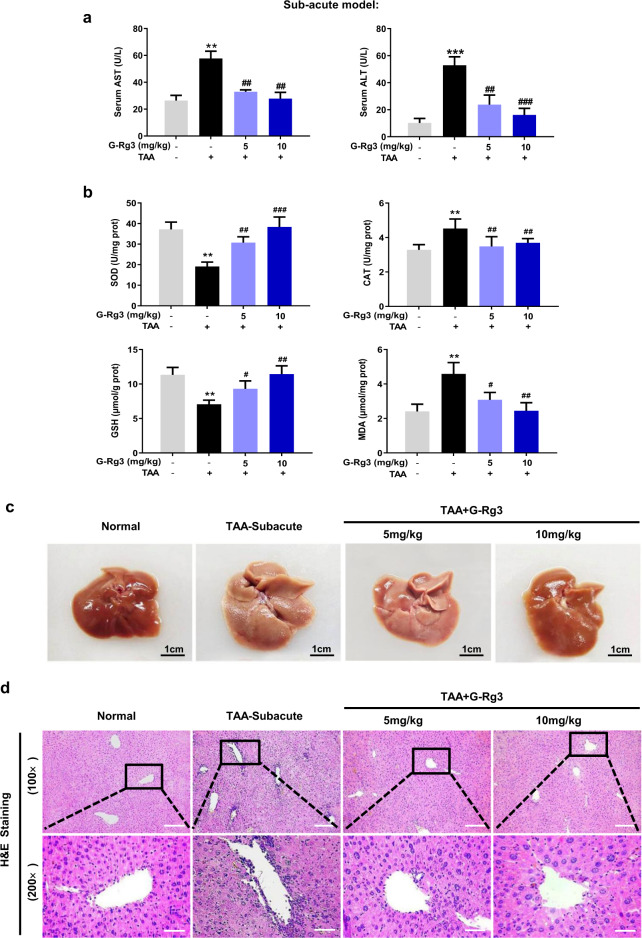
G-Rg3 ameliorated TAA-induced subacute hepatic injury in mice. Serological and histopathological examinations were performed with appropriate commercial kits. **a** Serum level of AST and ALT were tested by biochemical kit. **b** Oxidative stress levels were tested for SOD, CAT, GSH, and MDA in mice. **c** The difference in macroscopic pathological conditions of the liver in groups of Normal, TAA-Subacute model, TAA + G-Rg3 (5 mg/kg) and TAA + G-Rg3 (10 mg/kg) were shown after sacrifice. **d** Histopathological evaluation for liver damage. Representative images of liver sections of different groups stained with H&E staining with an amplification of ×100 and ×200. Values were shown as the mean ± S.D (*n* = 6); **p < 0.01, ***p < 0.001 *versus* Normal group; ^#^*p* < 0.05, ^##^*p* < 0.01, ^###^*p* < 0.001 versus TAA-Subacute group.

Following 4-weeks exposure of TAA, serological and histopathological examinations were performed with appropriate commercial kits. The serum levels of ALT and AST in the TAA-Subacute group were significantly higher than that in the control counterparts (Fig. 1a, ***p < 0.001). As illustrated in Fig. 1b, TAA-Subacute group showed significantly decreased levels of SOD and GSH, a nonsignificant slight decrease in CAT level, and an increase in the content of MDA. These changes indicated that TAA exerted severe damage on antioxidant defense system. TAA exerted considerable attack at a dose of 150 mg/kg in the TAA-subacute group, which finally induced lesion characteristics in the liver with whitening and porous surface (Fig. 1c). As shown in Fig. 1d, severe liver damage was caused by TAA with apparent parenchymal injury, inflammatory portal infiltration, accompanied by abundant inflammatory cells recruited around the hepatic portal area. The 2-weeks treatment with G-Rg3 (5 or 10 mg/kg) remarkably improved liver function with decreased levels of ALT and AST and reduced lipid peroxidation from that in TAA-induced subacute liver injury in mice (Fig. 1a, b).

**Fig. 2:**
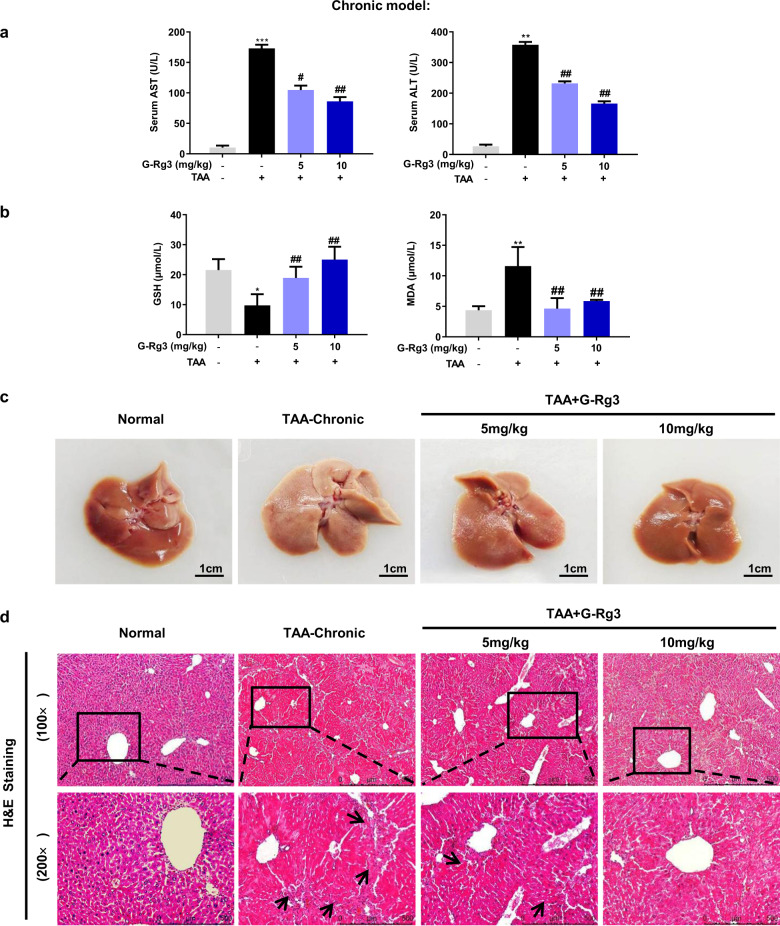
G-Rg3 ameliorated TAA-induced chronic hepatic injury in mice. Serological and histopathological examinations were performed with appropriate commercial kits. **a** Serum level of AST and ALT were tested by biochemical kit. **b** Oxidative stress levels were tested for GSH and MDA in mice. **c** The difference in macroscopic pathological observation of liver in groups of Normal, TAA-chronic model, TAA + G-Rg3 (5 mg/kg) and TAA + G-Rg3 (10 mg/kg) were shown after sacrifice. **d** Histopathological evaluation for liver damage. Representative images of liver sections of different groups stained with H&E staining with amplification of ×100 and ×200. Black arrows indicated long fiber interval. Values were shown as the mean ± S.D (*n* ≥ 8); *p < 0.05, **p < 0.01, ***p < 0.01 versus Normal group; ^#^*p* < 0.05, ^##^*p* < 0.01, *versus* TAA-chronic group.

In order to confirm TAA-induced inflammatory infiltration in the liver, 10-weeks observation was conducted with repeated stimulation. Long-term and repeated injections of TAA caused noticeable increase in liver pathological index, and raised relative spleen pathological index (Table 1). Meanwhile, TAA exerted no apparent effects in other organs. This result was confirmed by H&E staining (Fig. S1a). As shown in Fig. 2a, the levels of ALT and AST were dramatically elevated in the TAA-chronic group (for 10 weeks) indicating more severe liver damage than TAA-induced subacute hepatic injury (for 4 weeks). Compared to oxidative stress level in the TAA-subacute model, chronic TAA attack at 50 mg/kg caused similar depletion of GSH and elevation of the MDA level (Fig. 2b). These changes in GSH and MDA indicated that chronic stimulus increased hepatocyte lipid peroxidation, which contributed to the inflammatory cell infiltration. The pathological observation clearly showed hepatic morphological lesion with large areas of hepatocyte necrosis and increased inflammatory cells around the hepatic portal and sinusoids, followed by long fiber formation observed under H&E staining (Fig. 2c, d). Notably, there were no visible pathological changes in other organs such as heart, kidney, thymus, and duodenum but spleen (Fig. S1a), showing that TAA interfered metabolism process in liver and caused sustained inflammation infiltration in TAA-subacute and TAA-chronic models.

**Table 1:** Effects of G-Rg3 on body weights and organ weights of mice in TAA-induced chronic hepatic fibrosis model.

	Dosage	Body weights (g)		Organ indices (mg/g, ×100)		
Group	(mg/kg)	Final	Liver	Heart	Spleen	Kidney
Normal	**–**	44.25 ± 8.48	39.77 ± 2.20	4.80 ± 0.43	4.08 ± 0.95	14.74 ± 1.36
TAA-chronic	–	46.37 ± 3.57	50.50 ± 4.71^***^	4.71 ± 0.39	8.72 ± 4.26^*^	15.06 ± 2.02
TAA + Rg3(5 mg/kg)	50	47.19 ± 4.48	39.46 ± 5.06^###^	4.36 ± 0.33	4.76 ± 1.56	13.43 ± 1.41
TAA + Rg3(10 mg/kg)	50	47.29 ± 3.54	41.04 ± 1.77^###^	4.83 ± 0.47	4.03 ± 0.56^##^	15.47 ± 1.01

Values are expressed as the Mean ± S.D., *n* = 10.

*p < 0.05, ***p < 0.001 *versus* Normal group; ^##^*p* < 0.01, ^###^*p* < 0.001 *versus* TAA-chronic group.

### G-Rg3 effectively reversed hepatic fibrosis both in TAA-treated mice and HSC-T6 cells

**Fig. 3:**
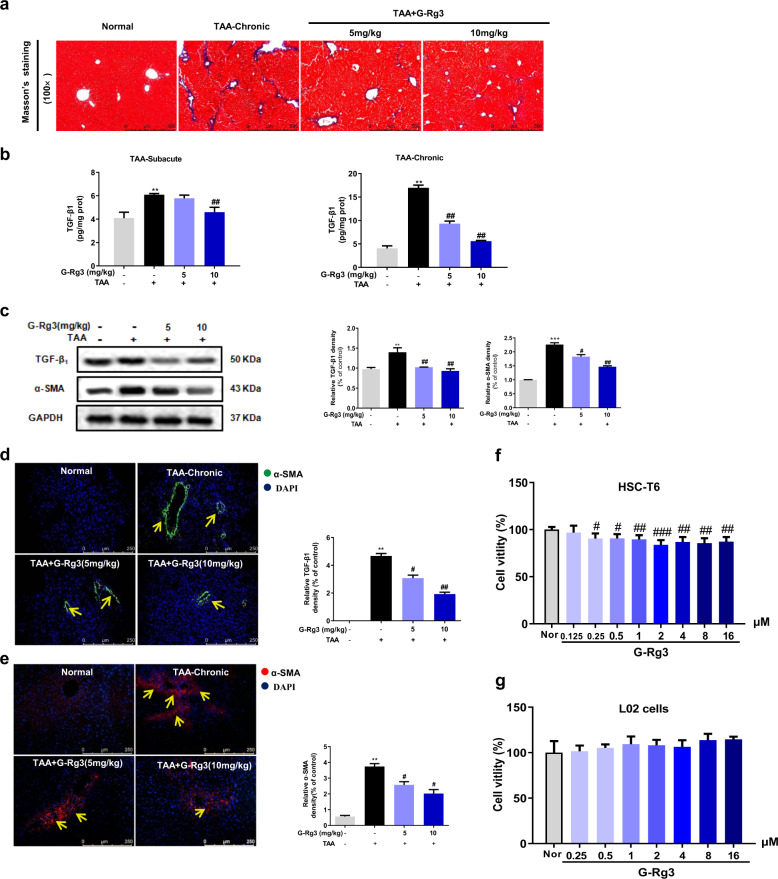
G-Rg3 effectively reversed TAA-induced chronic hepatic fibrosis through inhibition of HSCs activation in TAA-chronic model and in cells. **a** Masson’s staining was carried out to assess the situation of collagen deposition with an amplification of ×100 in TAA-chronic model. **b** Fibrogenic factor TGF-β1 was detected in serum level in both subacute hepatic injury models and chronic hepatic fibrosis models. **c** Expressions of TGF-β1 and α-SMA in chronic hepatic fibrosis model were detected by western blot analysis. Immunofluorescence staining for TGF-β1 (**d**) and α-SMA (**e**) were further carried out in TAA-chronic model with magnification of ×200, yellow arrows indicated TGF-β1-positive expression and α-SMA-positive cells, respectively. Effect of G-Rg3 on cell viability in HSC-T6 cells (**f**) and L02 cells (**g**). Values were shown as the mean ± S.D (*n* = 8). **p < 0.01, ***p < 0.001 *versus* Normal group; ^#^*p* < 0.05, ^##^*p* < 0.01, ^###^*p* < 0.001 *versus* TAA-chronic group in mice. ^#^*p* < 0.05, ^##^*p* < 0.01, ^###^*p* < 0.001 *versus* Normal group in cells.

To investigate the progression of hepatic fibrosis in mice, collagen deposition, HSCs activation marker α-SMA, and profibrogenic factor TGF-β1 were further measured. Masson’s staining (Fig. 3a) showed a significant increase in collagen deposition around periportal areas with marked portal-portal and portal-central bridging in TAA-Chronic group (for 10 weeks). Four-weeks oral administration of G-Rg3 (5 or 10 mg/kg) decreased collagen deposition and effectively reversed hepatic fibrosis. Furthermore, mice in TAA-induced subacute hepatic injury model (for 4 weeks) secreted more TGF-β1 than theirs in control group. There was a difference that TGF-β1 was clearly elevated in the long-term chronic TAA treatment group (Fig. 3b). Simultaneously, the mice in the TAA-chronic group showed increased α-SMA-positive staining (Fig. 3e) compared to the control mice. Western blot analysis and immunofluorescence staining (Fig. 3c, e) also confirmed the increase in α-SMA. Administration of G-Rg3 (5 or 10 mg/kg) effectively decreased the secretion of TGF-β1 and α-SMA expression, indicating an improvement of hepatic fibrosis. Hence, G-Rg3 prevented TAA-induced hepatic fibrosis in mice through inhibiting HSCs activation.

Reducing HSCs’ activation and cell proliferation serves an anti-fibrogenic index, which was used for screening anti-fibrotic activity of natural medicines^30,31^. The effects of G-Rg3 on HSC-T6 cells and L02 cells were tested in vitro. Pretreatment with G-Rg3 for 24 h (2–16 μM) inhibited the cell viability of HSC-T6 but not L02 cells (Fig. 3g). These results partially confirmed our conjecture that G-Rg3 diminished the viability of HSCs to control hepatic fibrosis progression and reversed TAA-induced hepatic fibrosis.

### G-Rg3 exerted anti-fibrotic effect through inhibiting LPS-induced autophagy flux

**Fig. 4:**
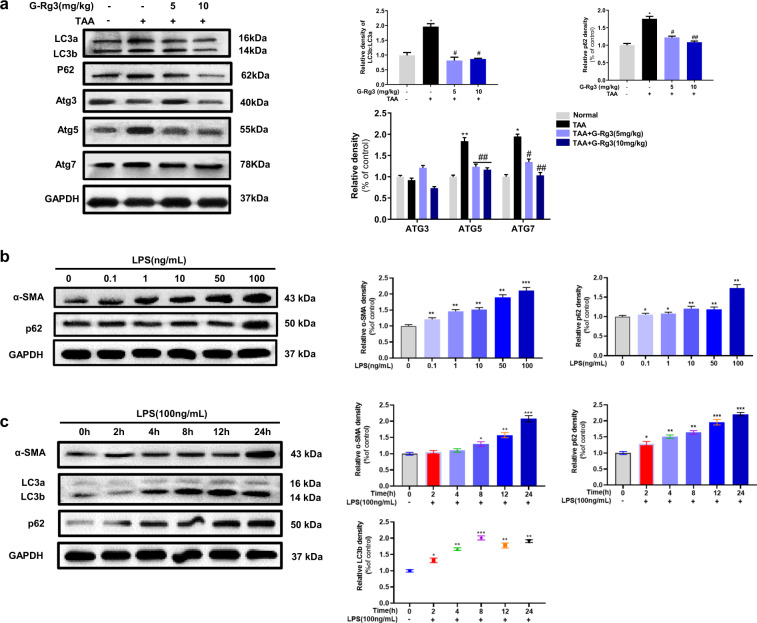

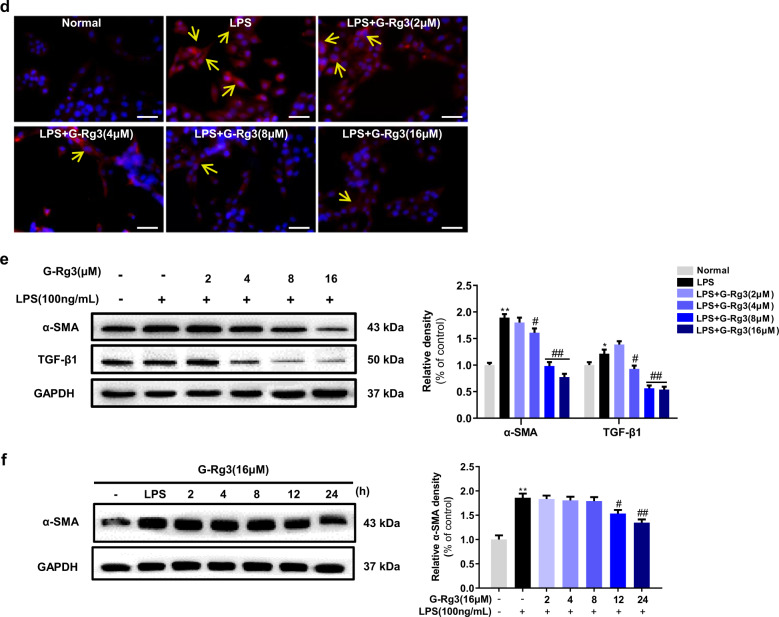
G-Rg3 ameliorated hepatic fibrosis through inhibiting autophagy flux both in vivo and in vitro. **a** Effect of G-Rg3 on autophagy-related proteins in TAA-Chronic model. The ratio of LC3b/LC3a represented an ignition of autophagy flux. Then, LPS-induced autophagy model was established in HSC-T6 cells. **b** Experiments for investigation of LPS doses (0–100 ng/mL) by western blot and normalized by GAPDH. **c** Evaluations of LPS treatment at 0, 2, 4, 8, 12, and 24 h were measured by expressions of α-SMA, LC3 a/b, p62. **d** Immunofluorescence staining of α-SMA with different concentrations of G-Rg3 incubation with an amplification of ×400. **e** Time of G-Rg3 treatment at 0, 2, 4, 8, 12, and 24 h were detected by western blot. **f** Expressions of TGF-β1 and α-SMA in HSC-T6 cells with G-Rg3 treatment. Values were shown as the mean ± S.D; *p < 0.05, **p < 0.01, ***p < 0.001 *versus* Normal group; ^#^*p* < 0.05, ^##^*p* < 0.01 *versus* TAA-chronic group, respectively.

Considering the association between fibrogenic factor TGF-β1 and autophagy, we further investigated the involvement of autophagy in TAA-induced chronic hepatic fibrosis in mice. As shown in Fig. 4a, ATGs proteins (ATG3, ATG5, and ATG7) expressed more in chronic TAA groups than that in control group, demonstrating that chronic TAA exposure triggered autophagy flux with more LC3a transformation into LC3b. Treatment with G-Rg3 partially decreased the ratio of LC3 b/a and the expression of p62. These data indicated that autophagy flux was partially reduced in vivo by the treatment of G-Rg3.

Inflammatory inducer LPS has been shown to induce autophagy in the liver hepatocytes and HSCs^32–34^. We firstly established in vitro trials on HSC-T6 cells by LPS stimulus. Among the concentrations of 50, 100, and 200 ng/mL, LPS induced a morphological variation of HSC-T6 cells, and no apparent changes occurred in the expressions of α-SMA and autophagy marker LC3 a/b when LPS was above 100 ng/mL (Fig. S2a, b). As shown in Fig. 4b, LPS treatment at 100 ng/mL activated HSC-T6 with increased expression of α-SMA, and increased expression of p62. The increased positive expressions of α-SMA in activated HSC-T6 cells were demonstrated by the immunofluorescence analysis (Fig. S2c). The changes in expression of LC3 a/b reached a peak at 8 h and kept stable ratio from 12 to 24 h, which activated HSC-T6 cells with a distinct expression of α-SMA at 24 h (Fig. 4c). Finally, 100 ng/mL of LPS incubation for 24 h induced obvious autophagy in HSC-T6 cells.

As depicted in Fig. 4d, pretreatment with G-Rg3 dose-dependently decreased LPS-induced positive expression of α-SMA in HSC-T6 cells, which was consistent with the results obtained from western blot analysis (Fig. 4e). Notably, there was a significant decline in TGF-β1 expression after G-Rg3 pretreatment, which supported the opinion that increased secretion of TGF-β1 partially originated from HSCs. G-Rg3 pretreatment at 12 h inhibited HSC-T6 cell activation with a decline in the expression of α-SMA up to 24 h (Fig. 4f). Moreover, the expressions of Beclin-1 and phosphorylated ULK1 were increased in the LPS group (Fig. S2d), indicating the initiation of autophagy. The increased expressions of Atg7 and Atg3 assisted the conversion of LC3a to LC3b on the surface of autophagic vesicles as pre-autophagosomes. As shown in Fig. S2d, the expressions of ATG5, ATG12, and ATG16L were increased after LPS stimulus, which formed multimer Atg12-Atg5-Atg16 through ubiquitin-like reaction to facilitate the expansion of autophagosomes. Pretreatment of G-Rg3 decreased the activation of HSC-T6 in a dose-dependent manner with less expression of α-SMA and profibrogenic factor TGF-β1 and reduced autophagy-related proteins, where TGF-β1 promoted the occurrence of autophagy.

### G-Rg3 inhibited Rapamycin-induced autophagy flux

**Fig. 5:**
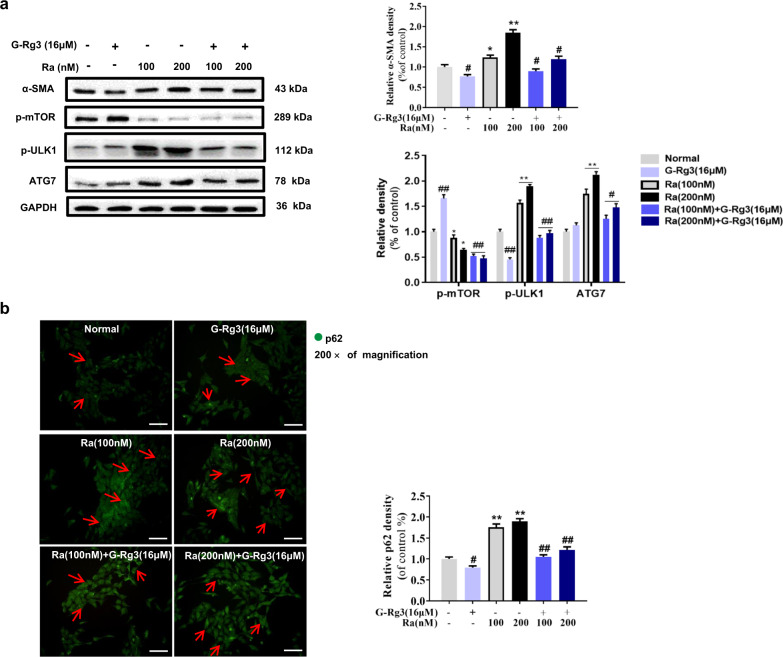
G-Rg3 inhibited Rapamycin-induced autophagy flux in vitro. **a** Autophagy was triggered by Rapamycin (Ra) at 100 or 200 nM in HSC-T6 cells with G-Rg3 pretreatment, and autophagy-related proteins were detected by western blot. **b** Immunofluorescence staining of p62 was conducted in HSC-T6 cells. G-Rg3 pretreatment with 16 μM and Ra were conducted with 100 or 200 nM. Relative fluorescence intensity was quantified. Red arrows indicated p62-positive cytoplasm expression and its location in HSC-T6 cells. Values were shown as the mean ± S.D; *p < 0.05, **p < 0.01 *versus* Normal group; ^#^*p* < 0.05, ^##^*p* < 0.01 *versus* Ra group, respectively.

To further testify the role of autophagy in the anti-fibrotic effect of G-Rg3, Rapamycin (Ra, 100 and 200 nM) was used to establish the autophagy activation model in HSC-T6 cells. Ra dramatically blocked the phosphorylation of mTOR at Ser2448 and decreased the expression of p-mTOR, which further activated autophagy-related proteins such as ATG7 and p-ULK1 at Ser757, and caused autophagy flux in HSC-T6 cells (Fig. 5a). Pretreatment with G-Rg3 partially reversed the changes of autophagy-related protein expression and α-SMA expression in HSC-T6 cells, i.e., less HSC-T6 cells activated. Persuasively, autophagy cargo protein p62 in Ra groups accumulated and distributed in the cytoplasm evidenced by immunofluorescence staining (Fig. 5b). Rapamycin at 100 nM initiated autophagy and pretreatment with G-Rg3 attenuated the autophagy reaction. Therefore, G-Rg3 intervenes the progression of autophagy and inhibits survival of HSC-T6 cells in vitro.

### PI3K/Akt-signaling pathway participated autophagy-flux-mediated hepatic fibrosis in vivo and in vitro

**Fig. 6:**
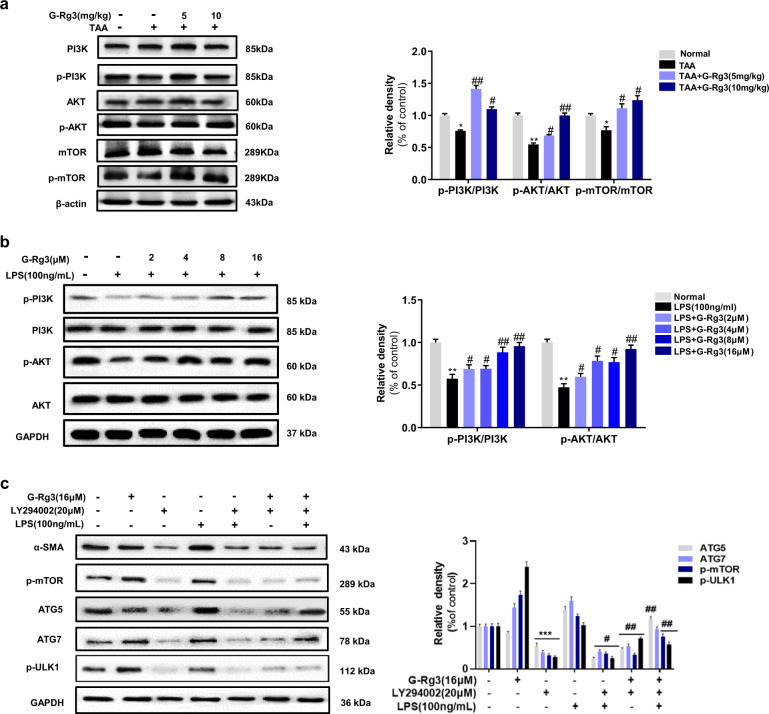
Regulation of PI3K/Akt on autophagy signaling pathway, both in chronic fibrosis model and HSC-T6 cells. **a** The expressions of PI3K, Akt, and mTOR were detected by western blot and all their bands were standardized by β-actin. **b** The expressions of PI3K, Akt were detected for LPS-induced HSC-T6 cells activation. **c** LY294002 (PI3K inhibitor) was used for LPS-induced autophagy in HSC-T6 cells. Relative density of α-SMA, p-PI3K, p-Akt, and p-mTOR were quantified with gel analysis system. Values were shown as the mean ± S.D; *p < 0.05, **p < 0.01, ***p < 0.001 *versus* Normal group; ^#^*p* < 0.05, ^##^*p* < 0.01 *versus* TAA-chronic group or LPS group.

The effect of G-Rg3 might be related to PI3K/Akt signaling owing to its well-known regulation on cell survival and proliferation. As depicted in Fig. 6a, the phosphorylation of PI3K and Akt decreased after chronic TAA exposure, and treatment with G-Rg3 reversed these changes. G-Rg3 effectively activated PI3K/Akt-signaling cascade in mice liver. Meanwhile, negative autophagy regulator mTOR phosphorylated more in G-Rg3 groups than those in the TAA-chronic group, which indicated inhibition of autophagy by G-Rg3 administration. Moreover, effect of G-Rg3 also verified involvement of PI3K/Akt-signaling pathway in autophagy. The results in HSC-T6 cells also supported this conclusion (Fig. 6b).

PI3K inhibitor LY294002 was used to explore further upstream signaling of autophagy and the role of PI3K in activated HSC-T6 cells. LY294002 (20 μM) was applied on HSC-T6 cells after pretreatment with 16-μM G-Rg3 (Fig. 6c). LY294002 inhibited HSC-T6 cell activation with a significant reduction in α-SMA and severely suppressed phosphorylation of mTOR compared to other groups. This inhibition was due to cytotoxic effects of LY294002 or non-specific PI3K inhibitor on autophagy, which may need further investigation. The autophagy-related protein such as ATG5, ATG7, and phosphorylated mTOR were all severely suppressed in LY294002 + G-Rg3 group or LPS + G-Rg3 group than in LY294002 + LPS + G-Rg3 group indicating that the suppression was induced by LY294002. This result demonstrated that G-Rg3 activated the Akt/mTOR-signaling pathway in LPS-induced hepatic fibrosis and autophagy in HSC-T6 cells. In summary, G-Rg3 inhibited the activation of HSCs which may be realized through reducing the progression of autophagy flux.

**Fig. 7:**
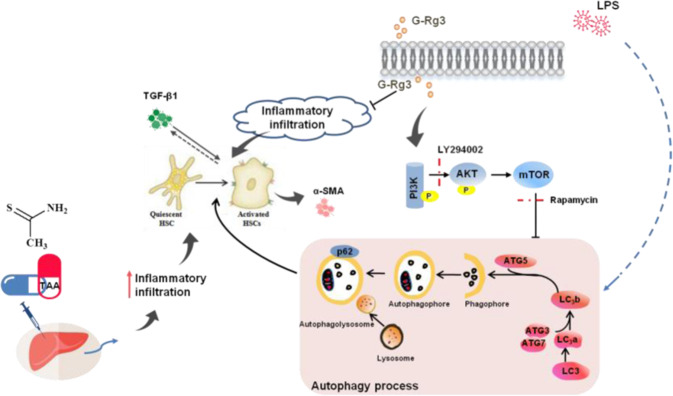
Molecular mechanism of G-Rg3 on signaling crosstalk in hepatic fibrosis models. G-Rg3 reversed TAA-induced hepatic fibrosis through decreasing the inflammatory infiltration in liver and inhibited HSCs' activation, which might be related to reducing inflammation-mediated autophagy signaling.

## Discussion

In this study, we revealed a novel method to model human hepatic cirrhosis in mouse and explored further the role of autophagy in liver fibrosis. Subacute and chronic TAA treatment models showed inflammatory infiltration in hepatocytes, and inflammatory response stimulated autophagy to aggravate fibrosis further. The inflammation inducer LPS was used to induce the occurrence of autophagy in HSC-T6 cells in vitro. G-Rg3 exerted anti-inflammatory effect and inhibited HSC-T6 cell survival without cytotoxicity effect on healthy hepatocytes L02. Furthermore, the excellent hepatoprotective property of G-Rg3 may activate Akt/mTOR-signaling cascade in HSCs.

G-Rg3 reversed the inflammatory infiltration-oriented hepatic fibrosis. Inflammation is one of the most characteristic features of chronic liver diseases of viral, alcoholic, fatty, and autoimmune origin^1^, and fibrosis occurred in virtually all types of liver diseases^35^, Compared to classical hepatic fibrosis models by CCl4 and BDL treatments, TAA presented several advantages as following: (i) TAA treatment shows pathological features closer to clinical cirrhosis, explicated as extensive inflammatory infiltration, residual hepatocyte nodular regeneration, connective tissue hyperplasia, fibrous septum formation, and hypersplenism (a characteristic of splenomegaly) in the advanced stage of liver fibrosis^36^. (ii) The modified method for TAA-induced hepatic fibrosis is effective and suitable for clinical screening of potential anti-fibrotic drugs. (iii) For its metabolic process, biotransformation of TAA precedes oxidative damage of liver injury. The hepatotoxicity of TAA requires its biotransformation to the highly reactive metabolite TAA-S-dioxide by NADPH and microsomal CYP2E1, further initiating necrosis by covalently binding to liver macromolecules^37,38^. Hepatic necrosis may affect regeneration of hepatocytes, promote secretion of inflammatory factors and increase expression of α-SMA from activated HSCs, which leads to the appearance of long fibrous spaces and further triggers portal and sinusoidal fibrosis. Although the long-term exposure of TAA on other organs may be ignored, incresed spleen index in TAA-chronic group indicating a symptom of hypersplenism at the advanced stage of hepatic fibrosis. Hepatic fibrosis is a continuous wound healing response, in which inflammation also plays a crucial role in the promotion of liver regeneration and progression of hepatic fibrosis^39,40^. As such, chronic inflammation, at least partially through continuous secretion of TGF-β1, stimulates further activation of HSCs. In subacute or chronic models, intraperitoneal injection of TAA elevated the level of oxidative stress with increased glutathione depletion and severe accumulation of lipid peroxidation, consistent with others^27,41^. Notably, oxidative stress was not a determinant factor for hepatic fibrosis, and it might serve as a catalyst in primary period of hepatic fibrosis.

G-Rg3, as a unique natural compound, may serve as hepatoprotective agent for hepatic fibrosis. In this study, G-Rg3 alleviated TAA-induced lipid peroxidation and inflammation infiltration as well as the deposition of collagen fibers through inhibitory activation on HSCs and release of the profibrotic factor TGF-β1. G-Rg3 dose-dependently inhibited the survival of HSC-T6 cells but did not affect the survival of L02 cells. Zhou et al*.* found that 20(R)-G-Rg3 suppressed APAP-induced hepatocellular necrosis and apoptosis and exerted protection on liver injury via activating PI3K/Akt-signaling pathway^26^. G-Rg3 might kill HCC cell lines without any toxicity on normal hepatocytes^42^, which was similar to our results. Activation of PI3K/Akt-signaling cascade attenuated hepatic fibrosis, through regulation on hepatocytes or other types of cells^27,43^. In this study, G-Rg3 inhibited HSCs’ survival might be through activating PI3K/Akt cascade signal in vivo and in vitro. Undoubtedly, G-Rg3 regulates various cell survivals via different pathways. Besides, the inhibition of autophagy flux caused by G-Rg3 suggests that autophagy may play a role between G-Rg3 and the PI3K/Akt-signaling cascade under hepatic fibrosis.

Autophagy is recognized as an evolutionarily conserved process to recycle the damaged and dysfunctional cytoplasmic constituents, and usually activated by energy restriction, stress, or inflammation^44^. Ginsenosides have been proven to affect autophagy. For example, G-Rg3 inhibited the increased expression of autophagy flux in non-small-cell lung cancer, which might be related to the blockage for fusion of lysosomes and degradation of autophagic lysosomes^42,45^. In this research, an increase in autophagy flux was observed in TAA-induced chronic hepatic fibrosis. Here autophagy might be related to inflammatory stimulus. Besides, as a inflammatory inducer, LPS was increased in the liver cirrhotic^46^, and LPS-related endotoxemia aggravated hepatic fibrosis through triggering autophagy^32^. Yet, inhibition of autophagy suppressed HSCs activation and proliferation^47^. In the current research, LPS apparently induced cell morphology changes and cell activation of HSC-T6 cells, and resulted in the increase in ratio of LC3b/a as well as a high expression of p62. G-Rg3 pretreatment reduced these changes from 12 to 24 h treatments in a dose-dependence manner, which effectively against LPS-induced transient and long-term stimulus, similar to the effect in TAA-treated mice. Akt-mTOR cascade is considered a major negative regulator of autophagy^48^. mTOR inhibitor, Rapamycin, activated autophagy process with increased expression of p62. Whereas pretreatment of G-Rg3 partially attenuated the effects of Rapamycin with decreased activation of HSC-T6 cells. G-Rg3 activated PI3K/Akt-signaling pathway and inhibited autophagy flux in vivo, but PI3K might not be the specific target to regulate autophagy upstream signaling. PI3K inhibitor, LY294002, was not a highly selective PI3K blocker due to its effect outside of autophagy to cause apoptosis in treated cells. The Class III PI3K, Vps34, is required for the activation of mTOR^49^. Notably, the results of high expression of p62 in LPS group were different from results of others^50^. p62 protein may also be elevated in cancers^51^, and chronic p62 elevation contributes to HCC development through prevention of senescence and death of cancer-initiating cells with enhanced proliferation^52^. Robin et al. proposed that autophagy-defective tumor cells preferentially accumulated p62 in response to stress^53^. Furthermore, p62 is a stress-induced protein occupying a central position in many signaling systems in cell stress with deficient autophagic clearance. Moreover, many studies have shown that autophagy promotes the deterioration of liver fibrosis, but fails to find a particular target site to treat hepatic fibrosis.

In summary, current experiments have unraveled that G-Rg3 may serve as a novel autophagy inhibitor and as an effective hepatoprotective drug in the early stage of hepatic fibrosis.

## Supplementary information

Supplement Figure Legends

Figure-Supplement1

Figure-Supplement2

Figure-Supplement3
