# Supplementary material for: Ginsenoside Rg3 promotes regression from hepatic fibrosis through reducing inflammation-mediated autophagy signaling pathway
Source: Cell Death Dis. 2020 Jun 12;11(6):454. doi: 10.1038/s41419-020-2597-7 (PMC7293224; doi:10.1038/s41419-020-2597-7)
Supplement: Supplementary file 1 — Supplement Figure Legends [file 41419_2020_2597_MOESM1_ESM.docx]

***Supplement Figure legends***

**Figure S1.** Pathological observations were conducted on spleen, heart, duodenum, kidney, and thymus in TAA-Chronic model, with an amplification of 100 × and 200 ×.

**Figure S2.** Autophagy-related experiments in vitro. (A) Representative images for morphological observation of LPS stimulated HSC-T6 cells with an amplification of 400 ×. (B) Experiments for investigation of LPS doses (0-200 ng/mL) by western blot and normalized by GAPDH. (C) Immunofluorescence staining of α-SMA with various concentrations of LPS with an amplification of 400 ×. (D) Expressions of autophagy-related proteins in LPS-treated HSC-T6 cells were detected by western blot and normalized by GAPDH.
