# Supplementary figures and images for: Ginsenoside Rg3 promotes regression from hepatic fibrosis through reducing inflammation-mediated autophagy signaling pathway
Source: Cell Death Dis. 2020 Jun 12;11(6):454. doi: 10.1038/s41419-020-2597-7 (PMC7293224; doi:10.1038/s41419-020-2597-7)

Figure-Supplement1

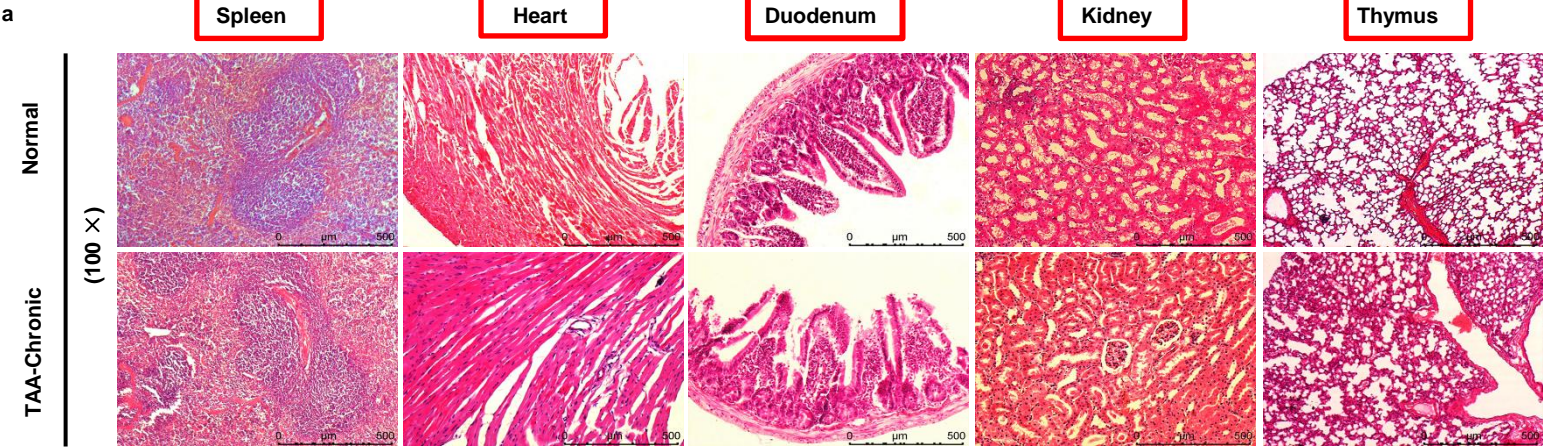

Supplement: Supplementary file 2 — Figure-Supplement1 [file 41419_2020_2597_MOESM2_ESM.pdf]

a

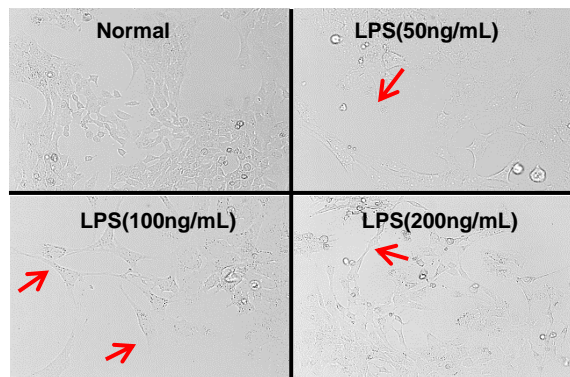

b

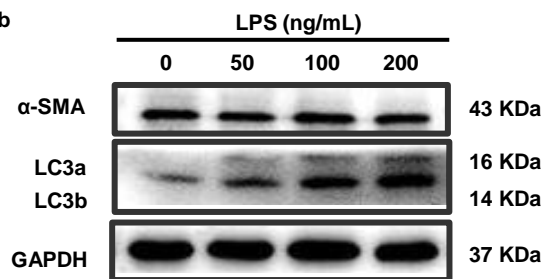

c

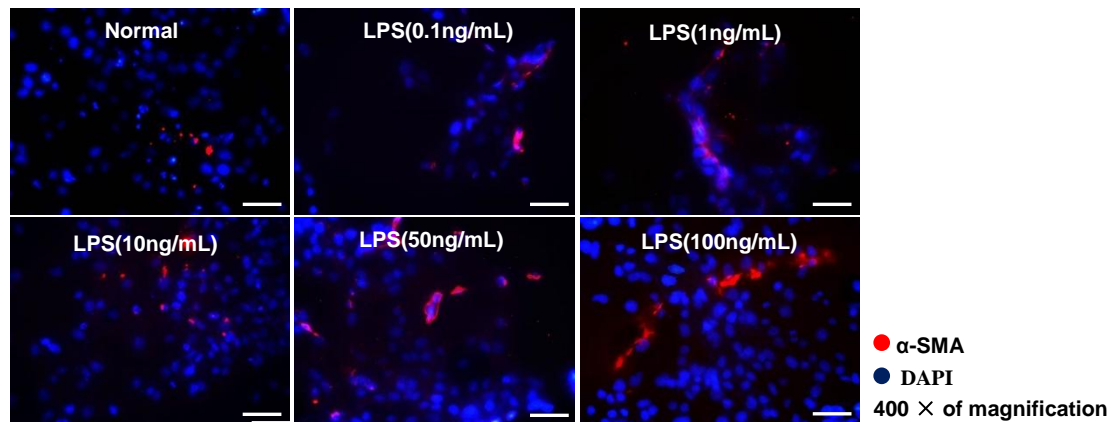

Supplement: Supplementary file 3 — Figure-Supplement2 [file 41419_2020_2597_MOESM3_ESM.pdf]

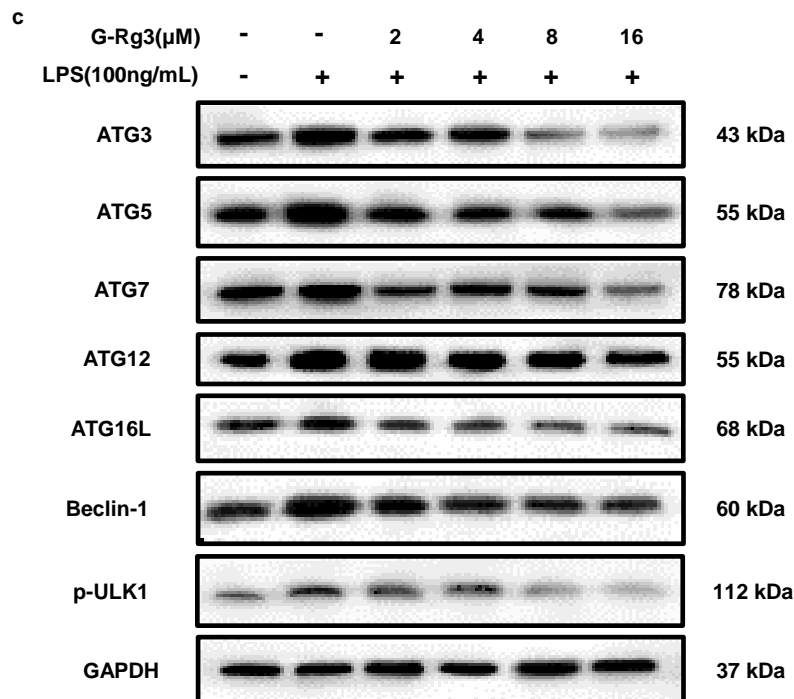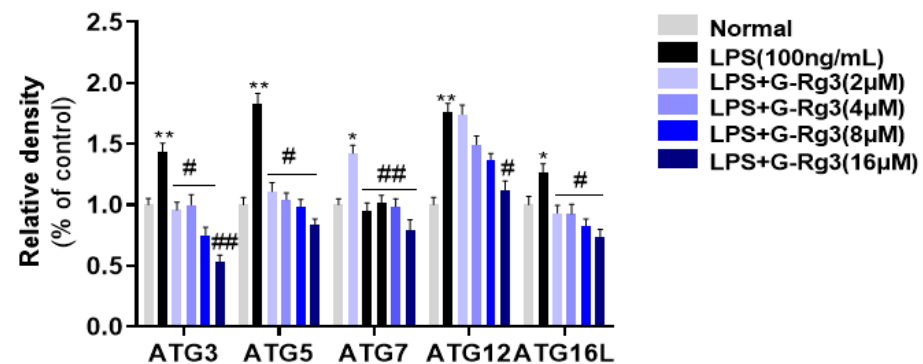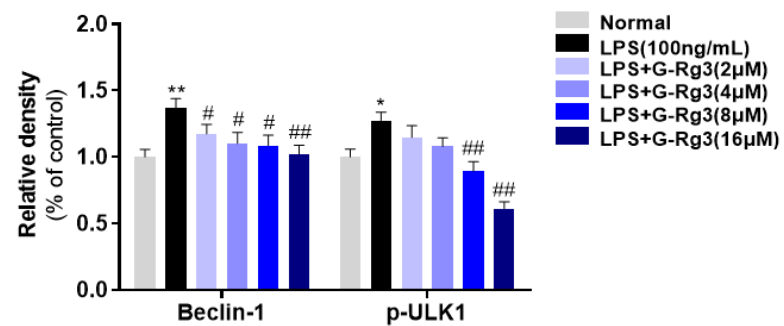

Supplement: Supplementary file 4 — Figure-Supplement3 [file 41419_2020_2597_MOESM4_ESM.pdf]
